# SDAMPP report #1: Holistic review for medical physics residency applicant selection

**DOI:** 10.1002/acm2.70649

**Published:** 2026-06-04

**Authors:** Amy Yu, Abby Besemer, Courtney Buckey, Ashley Cetnar, Lindsay DeWeese, Yu Gao, Patricia Lindsay, Anna Rodrigues, Leah Schubert, Joshua Wilson, Sarah Wisnoskie, Titania Juang

**Affiliations:** ^1^ Department of Radiation Oncology Stanford University Cancer Center Stanford California USA; ^2^ Department of Radiation Oncology University of Wisconsin Madison Madison Wisconsin USA; ^3^ Department of Radiation Oncology Mayo Clinic Arizona Phoenix Arizona USA; ^4^ Department of Radiation Oncology The Ohio State University – James Cancer Hospital Columbus Ohio USA; ^5^ Department of Diagnostic Radiology Oregon Health & Science University Portland Oregon USA; ^6^ Department of Radiation Oncology Stanford University Palo Alto California USA; ^7^ The Princess Margaret Cancer Centre – UHN Toronto Ontario Canada; ^8^ Department of Radiation Oncology Duke University Medical Center Durham North Carolina USA; ^9^ Department of Radiation Oncology University of Colorado Denver Aurora Colorado USA; ^10^ Department of Radiology Duke University Health System Durham North Carolina USA; ^11^ Physics Novant Health Winston Salem North Carolina USA; ^12^ Radiation Medicine & Applied Sciences UC San Diego San Diego California USA

**Keywords:** attributes, competencies, experiences, holistic review, metrics, residency applicant selection

## Abstract

Clinical medical physics is a profession that intersects with various groups as part of a broader healthcare team, including dosimetrists, radiation therapists, radiologic and MRI technologists, IT specialists, and physicians. Success in this field requires academic and technical excellence as well as comprehensive skills for working effectively within an interdisciplinary healthcare team. When hiring medical physics residents, the selection of well‐rounded and highly qualified candidates can be facilitated through a holistic review process that ensures consideration of traditional metrics such as academic performance while also accounting for the importance of various experiences, personal attributes, and intrinsic motivations that impact clinical performance and patient care. The Association of American Medical Colleges (AAMC) emphasizes an overall consideration of Experiences, Attributes, Competencies, and Metrics (EACM) as important for the search process. As part of the healthcare community, medical physics shares the same commitment to high‐quality patient care. By adopting the AAMC's EACM framework, medical physics residency programs can strengthen the fairness, rigor, and overall effectiveness of their application review processes—ultimately benefiting applicants, the medical physics community, and the patients we serve. This Society of Directors of Academic Medical Physics Programs (SDAMPP) task report aims to provide information and resources for applying holistic review to the applicant selection process for medical physics residency programs. Our objective is to enable program directors to reevaluate their selection methods in the context of institutional and program missions and goals and to improve the quality and specificity of recruitment by integrating holistic review concepts into their residency recruitment processes. This initiative can inspire change in recruitment practices, aid programs in hiring well‐rounded and well‐qualified residents, and support the professional growth of our trainees in healthcare. This report provides an overview of holistic reviews and its core principles. We review the current literature and provide evidence from successful implementations of holistic review in other medical fields to illustrate how it can benefit our field. Furthermore, we outline actionable recommendations to facilitate transitioning to a holistic review framework and address potential barriers with practical solutions. A supplemental workbook is provided to guide residency programs in developing their own holistic application review and interview processes. With this report, we aim to provide practical implementation guidelines that will allow residency program directors to implement a selection methodology that comprehensively assesses the full qualifications of individual candidates and ultimately fosters the development of versatile and competent clinical medical physicists who are best equipped to advance the field, meet patient needs, and improve patient care.

## 1 FOREWORD

Clinical medical physics is a profession that intersects with various groups as part of a broader healthcare team, including dosimetrists, radiation therapists, radiologic and MRI technologists, IT specialists, and physicians. Success in this field requires academic and technical excellence as well as comprehensive skills for working effectively within an interdisciplinary healthcare team. When hiring medical physics residents, the selection of well‐rounded and highly qualified candidates can be facilitated through a holistic review process that ensures consideration of traditional metrics such as academic performance while also accounting for the importance of various experiences, personal attributes, and intrinsic motivations that impact clinical performance and patient care. The Association of American Medical Colleges (AAMC) emphasizes an overall consideration of Experiences, Attributes, Competencies, and Metrics (EACM) as important for the search process. As part of the healthcare community, medical physics shares the same commitment to high‐quality patient care. By adopting the AAMC's EACM framework, medical physics residency programs can strengthen the fairness, rigor, and overall effectiveness of their application review processes—ultimately benefiting applicants, the medical physics community, and the patients we serve.

This Society of Directors of Academic Medical Physics Programs (SDAMPP) task report aims to provide information and resources for applying holistic review to the applicant selection process for medical physics residency programs. Our objective is to enable program directors to reevaluate their selection methods in the context of institutional and program missions and goals and to improve the quality and specificity of recruitment by integrating holistic review concepts into their residency recruitment processes. This initiative can inspire change in recruitment practices, aid programs in hiring well‐rounded and well‐qualified residents, and support the professional growth of our trainees in healthcare.

This report provides an overview of holistic reviews and its core principles. We review the current literature and provide evidence from successful implementations of holistic review in other medical fields to illustrate how it can benefit our field. Furthermore, we outline actionable recommendations to facilitate transitioning to a holistic review framework and address potential barriers with practical solutions. A supplemental workbook is provided to guide residency programs in developing their own holistic application review and interview processes. With this report, we aim to provide practical implementation guidelines that will allow residency program directors to implement a selection methodology that comprehensively assesses the full qualifications of individual candidates and ultimately fosters the development of versatile and competent clinical medical physicists who are best equipped to advance the field, meet patient needs, and improve patient care.

## 2 DEFINITIONS

*Application review*: The processes used by residency programs to evaluate the applications submitted by prospective residents. The goal of the *application review* is to identify candidates who exhibit the necessary qualifications, skills, and qualities to succeed in the residency program.

*Screening interview*: The initial interview conducted by some residency programs to assess candidates' qualifications for the program. It is conducted before inviting prospective residents for the *final interview*.

*Final interview*: The last step in the interview process for prospective residents. This interview is more in‐depth and may involve multiple faculty members and staff from the residency program.

*Experiences*: The path that applicants have taken to get where they are and the context in which these experiences have taken place.

*Attributes*: Applicants’ skills, abilities, qualities, and relevant background. This might include characteristics like intellectual curiosity, cultural humility, or proficiency in more than one language.

*Competencies*: Ways in which the applicant applies their knowledge, skills, and abilities to the job. These competencies ensure that medical professionals are equipped to provide high‐quality care and meet the specialty's demands.

*Metrics*: The quantitative scholarly and academic components of an applicant's portfolio.

*EACM model*: A framework used to evaluate candidates holistically using four key components: Experiences, Attributes, Competencies, and Metrics.

*Implementation committee*: A group of individuals assembled to use the holistic review framework to identify and prioritize criteria, create assessment rubrics, generate standardized interview questions, provide training, and generally oversee and evaluate the holistic review framework process over time.

*Selection committee*: A group of individuals who are typically responsible for performing the application review, screening interviews, final interviews, and ranking candidates during the residency recruitment process.

*Structured interviews*: A systematic method of candidate evaluation in which the interviewer follows a predetermined set of questions and a specific format. This approach ensures consistency across interviews, allowing for fair comparison and analysis of responses.

*Application blinding*: A process where the identities of applicants are concealed from the reviewers during the evaluation of their applications. This is done to reduce potential biases based on factors such as the applicant's name, gender, ethnicity, or academic institution name. The goal of application blinding is to promote fairness and equity in the selection process, allowing reviewers to focus solely on the qualifications, experiences, and competencies of the applicants without being influenced by personal characteristics unrelated to the position.

*Anchored rubric*: A structured assessment tool that provides specific criteria and descriptions of multiple performance levels for evaluating candidates in a comprehensive manner. This type of rubric is designed to ensure that the evaluation process is consistent, fair, and aligned with the values and competencies desired in a candidate.

## 3 INTRODUCTION

The role of the medical physicist is complex and continues to evolve to provide excellent patient care. Recommendations for residency training standards for specialties are provided by the American Association of Physicists in Medicine (AAPM) educational appointed groups,^1^, ^2^, ^3^ and programs are accredited by the Commission on Accreditation of Medical Physics Education Programs (CAMPEP).^4^ It is important to consider equitable recruitment methods for our profession, especially given the current residency training environment where there are more candidates than available residency positions. As of 2025, there are over 160 CAMPEP‐accredited residency programs, and although the number of medical physics residency programs continues to rise, this increase has been outpaced by the increase in applicants. This is reflected in data from the MedPhys Match, a centralized matching system used by the majority of medical physics residency programs to fill their open residency positions: according to statistics from an annual survey required of all CAMPEP programs, 71% of residency programs have indicated that they will participate in the MedPhys Match for some or all of their positions in 2025. Based on MedPhys Match Statistics, over the past five years, the numbers gap between applicants and available residency positions has widened significantly. While the number of residency positions in the MedPhys Match has grown by approximately 39% since 2022, the number of registered applicants has surged by 63%, creating an increasingly competitive environment.

While accrediting bodies provide requirements for what training aspects must be included in a program, each residency program has the important role to defining their individual mission and goals as a clinical training program within their respective institutions. This clarity and focus on the program's overarching principles theoretically define the qualities that make a successful resident for each unique program. Once explicitly established and agreed upon, assessment metrics can be determined to help guide a fair recruitment process to identify competent candidates who align with the values of the institution and the specific program.

A key aspect of program administration is conducting the recruitment and selection process. While traditional quantitative metrics, such as academic achievements, standardized test scores, and research accomplishments, are clearly defined and easily measurable, they provide only a partial picture of a candidate's potential. Many other essential attributes, such as interpersonal skills, resilience, leadership, and problem‐solving abilities, are harder to quantify but are critical for navigating complex clinical environments and fostering collaboration within multidisciplinary teams. Moreover, research has shown that a workforce with differing experiences and perspectives promotes innovation, strengthens problem‐solving abilities, and enhances cultural competence, all of which are essential for delivering high‐quality patient care to all populations.^5^, ^6^

**FIGURE 1:**
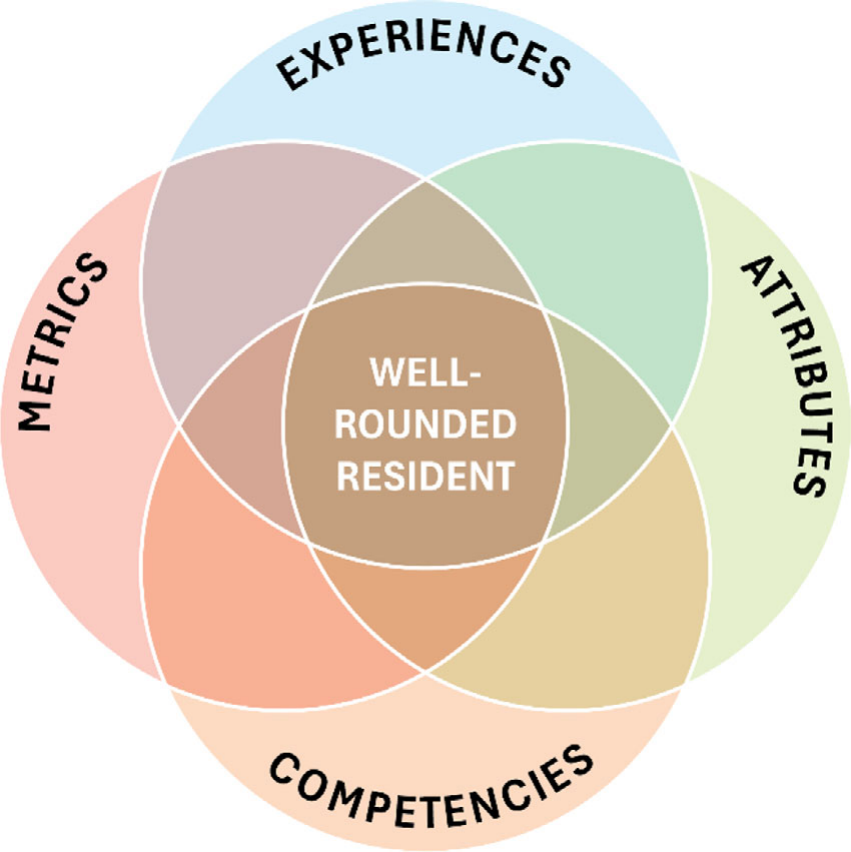
A framework used to evaluate candidates holistically using four key components, including experiences, attributes, competencies, and metrics (EACM), to identify a well‐rounded resident.

The holistic review process provides a framework to evaluate candidates in terms of their past Experiences, personal Attributes, current Competencies, and traditional quantitative Metrics (EACM^7^), Figure 1. The Association of American Medical Colleges (AAMC) defines holistic review as a flexible, individualized approach emphasizing balanced consideration of EACM tailored to program‐specific goals.^8^ For residency selection, it involves evaluating candidates based on a comprehensive assessment of their qualifications, including clinical experiences, academic achievements, personal attributes, and professional accomplishments, as well as other attributes and experiences that are specific to the position and overall mission of the program.^8^, ^9^, ^10^ The goal is to select individuals who not only excel academically but also demonstrate the personal qualities, interpersonal skills, and diverse experiences that contribute to their potential as effective and altruistic physicists.

While some paths are more linear than others, all candidates have unique journeys in their study of medical physics. A strong and dynamic workforce benefits from a range of perspectives, experiences, and ideas, which ultimately enhances innovation and problem‐solving in patient care. According to a white paper issued by the American Society for Radiation Oncology (ASTRO) Society of Chairs of Academic Radiation Oncology Programs, Association for Directors of Radiation Oncology Programs, and Association of Residents in Radiation (SCAROP‐ADROP‐ARRO), employing a multi‐axis approach to holistic review allows evaluators to assess various attributes at the same time, promoting fairness in evaluating a diverse group of applicants. This method enables a just comparison between candidates who have had abundant opportunities and made the most of them, and those who, despite facing limitations, have shown remarkable initiative and accomplishments.^9^ Initial screening techniques—such as application reviews and screening interviews—play a critical role in determining which applicants are seriously considered and which are filtered out early in the process. If not thoughtfully designed, these steps can unintentionally exclude strong candidates, and such losses are rarely reversible later in the process.^11^ Once an applicant is screened out, they are typically no longer in contention, making it essential that early‐stage evaluations are both equitable and inclusive. To support this, several evidence‐based strategies have been identified, including blinding components of the application to reduce bias, implementing standardized processes for screening, using structured interview questions to ensure consistency, and engaging reviewers from diverse professional roles and levels within the program.^7^, ^12^ These approaches help ensure that a broad and diverse pool of applicants remains in consideration throughout the review process.

The holistic review approach has been successfully implemented in various healthcare residency selection processes to create fair opportunities for all candidates and to minimize biases in the selection process. Aibana et al. standardized the interview process and incorporated life experiences and personal attributes beyond academic credentials to enhance applicant assessment in internal medicine residency selection.^13^ They also explicitly communicated their program goals to all candidates on interview days. These measures resulted in equitable opportunities for applicants and notably increased the diversity of experiences and perspectives among those reviewed, interviewed, and matriculated. In emergency medicine, Sungar et al. adopted a holistic review process using a revised application review rubric that balanced EACM,^11^ resulting in a more comprehensive and inclusive evaluation. Similarly, a comprehensive scoring system was introduced in urology residency selection, focusing on desirable attributes aligned with the program's training needs and resources.^14^ This holistic approach led to broader geographic representation among interviewees while maintaining consistent academic performance metrics compared to previous years.

Despite promising evidence and shared successes from programs that have adopted holistic review, a lack of clear, well‐defined guidelines specifying criteria, evaluation methods, and standardized practices remains a significant barrier in the field of medical physics. This gap may lead to inconsistent or biased evaluations that can compromise the objectivity and effectiveness of a holistic review. To support the wider adoption and successful implementation of holistic review, the development of standardized guidelines that can be tailored to each program's unique needs is urgently needed.

## 4 COMPONENTS OF HOLISTIC REVIEW

The EACM framework should be implemented with respect to a program's curriculum, mission, and goals to generate the appropriate selection criteria for each stage of the recruitment process, henceforth referred to as the *application review*, *screening interview*, and *final interview*. As such, the implementation of a holistic recruitment process requires a “ground‐up” approach prior to its initial implementation:

- Identification: Selection criteria for each EACM category shall be defined.
- Prioritization: The importance of each selection criterion shall be established.
- Temporalization: The stage(s) where selection criteria are evaluated in the recruitment process shall be established.

Example individual selection criteria for each EACM category are shown in Table 1.

**TABLE 1:** Example criteria for experiences, attributes, competencies, and metrics.

**Experiences** **Significant events, activities, or periods in an individual's life that have shaped perspectives, skills, and character**. Challenges overcome Breadth of life experience Job experience (non‐medical physics) Clinical medical physics experience Leadership roles Service to the community Teaching experience Educational pathway Research experience outside medical physics Professional development (e.g., workshops, conferences)	**Attributes** **Personal qualities, characteristics, or traits that an individual possesses**. Critical reasoning Attention to detail Growth mindset/Initiative/Intellectual curiosity Leadership skills Motivation for a medical physics career Reliability and dependability Emotional intelligence Respect for staff and patients Self‐motivated learner/Teachability Teamwork Interest in specialty (i.e., therapy or imaging) Interest in the institution/program Added value to the program Desire to teach
**Competencies** **Specific skills, knowledge, or abilities that an individual has developed to perform tasks, solve problems, or achieve goals**. Resilience and adaptability Problem solving Time management skills Ability to handle high‐stress situations Communication skills Strong work ethic Ability to handle disappointment Ability to handle feedback Ability to ask for help Professionalism Solid foundation of knowledge Conflict resolution Ability to work with a wide range of individuals Organizational skills	**Metrics** **Quantifiable measures or indicators that provide evidence of an individual's achievements, progress, or potential**. Medical Physics graduate degree (e.g., MS/PhD) Grants/patents Honors/awards Registered for or passed ABR Part 1 GPA Peer‐reviewed publications Proffered abstracts CAMPEP degree/certificate Conference presentations

*Note*: Some criteria may appear to fall under multiple categories within the EACM framework. The criteria shown in the table are examples to illustrate potential selection criteria for each EACM category. Implementation committees can determine which criteria fit best under each category, and are encouraged to develop criteria with greater specificity (e.g., specific skills that describe the broader competency examples listed in the table). Program directors can also refer to the examples provided by the AAMC, https://www.aamc.org/media/44586/download, to better understand how to categorize selection criteria under the EACM model.

After the selection criteria are identified, prioritized, and temporalized, each selection criterion needs to be clearly defined in terms of assessment and evaluation rubrics. The implementation of a holistic recruitment process leads to a structured recruitment process with clearly defined timelines and expectations, assessment, and evaluation rubrics for the recruitment committee. By establishing transparent criteria and standardized evaluations, this approach enhances accountability and fairness.

## 5 PRACTICAL IMPLEMENTATION OF HOLISTIC REVIEW

To implement a holistic review process for resident selection, several key steps must be taken before, during, and after the application review and interview phases. Each of these steps requires decisions to be made and adopted by the program faculty as a whole. This section discusses recommendations and specific tools to aid in implementing holistic review, including a newly developed holistic review workbook tool that can support this process by guiding programs through these steps.

In the preparation phase of a holistic applicant review process, the implementation committee will discuss which criteria are most important and the weight of each of the criteria. Following the initial preparatory activities and establishment of selection criteria by the program, the descriptions of the residency program and position(s) on all materials viewed by potential applicants should be reviewed and updated, if necessary. Additionally, internal documents (self‐study, etc.) should also be reviewed and updated. This is to ensure accurate representation of program values and transparent application processes across different sources, such as the program's website, job postings, and recruitment materials.

For the initial application review, one of the decisions to be made includes identifying the number of reviewers and specifically who will serve on the selection committee. Creating a knowledgeable selection committee with commitment, integrity, and a balance of perspectives will help to ensure a fair and effective evaluation process. Moreover, the incorporation of multiple application reviewers with a clear, anchored rubric will allow for a more impartial approach to evaluating residency candidates.

There are additional decisions that the program will need to make when designing their review and interview processes. Holistic review is compatible with a variety of interview sequences (adding screening interviews as a first phase of interviewing or conducting a single round of interviews) and the interview format (virtual vs. in‐person), the incorporation of an applicant presentation, the number of faculty in each interview session (individual vs. group), and the incorporation of staff outside of medical physics.

### 5.1 Workbook overview: Implementing holistic review

To guide programs through implementing the holistic review process, this report includes a comprehensive workbook. The workbook is divided into seven sections:

1. Preparation
2. Applicant Criteria Identification & Prioritization
  - Experiences
  - Attributes
  - Competencies
  - Metrics
3. Final Selection Criteria
4. Application Review
5. Screening Interview
6. Full Interview
7. Question Bank

Each section is designed to provide step‐by‐step instructions and tools to facilitate a structured and effective implementation process.

**FIGURE 2:**
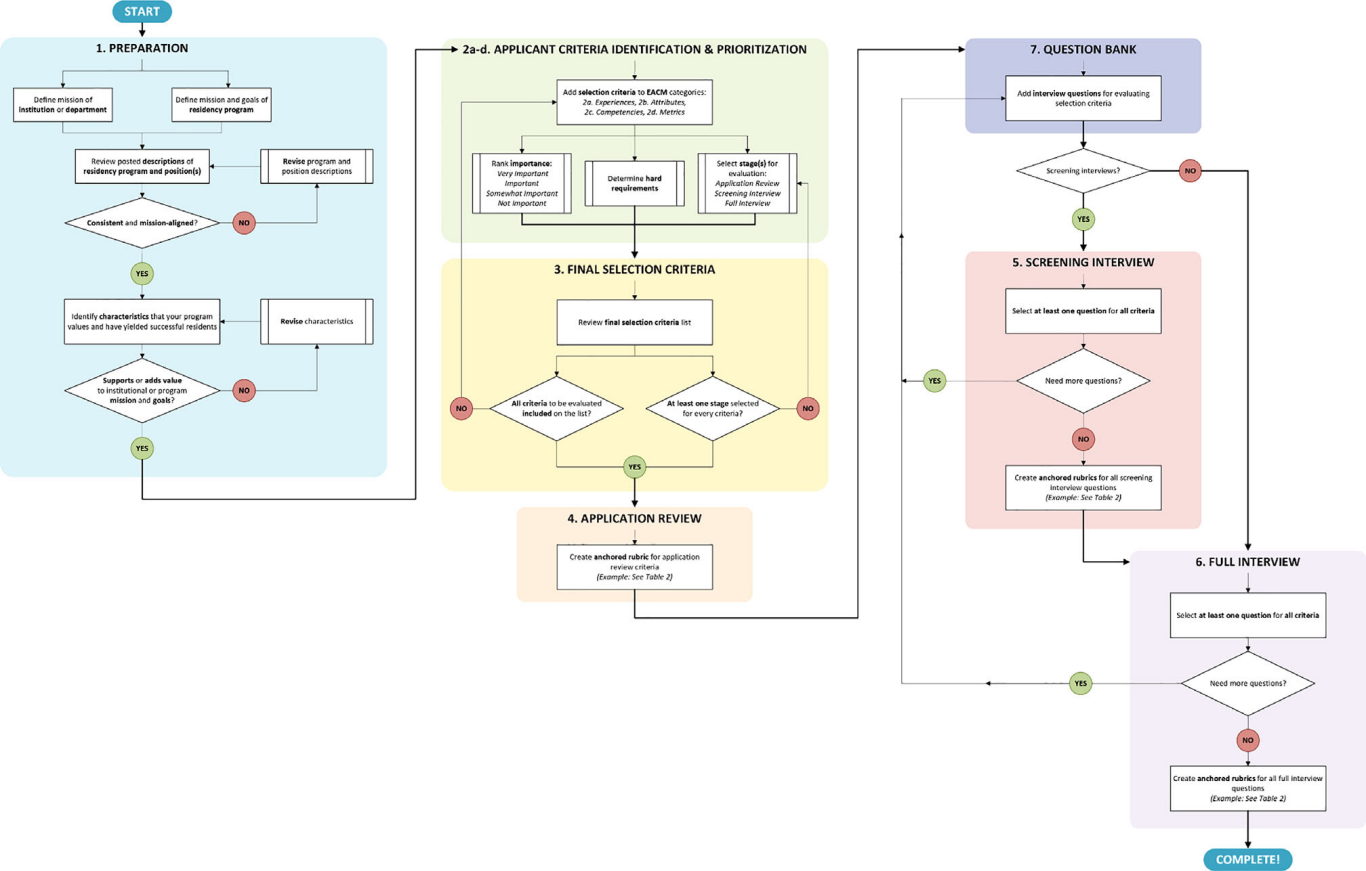
Medical physics holistic workbook flowchart.

A flowchart illustrating the workflow for the Medical Physics Holistic Review Workbook is shown in Figure 2. Readers can also find a recorded training video here (https://youtu.be/HvvYXyNlFTY) to orient themselves to the workbook functionality.

#### 5.1.1 Preparation

The preparation step ensures that the subsequent steps are tailored to a program's unique priorities and values. Working through the preparation helps program directors and the implementation committee complete essential pre‐work exercises to establish a foundation for the holistic review and recruitment process:

- **List institutional and program missions/goals**: Identify the mission and goals of the institution and/or department and of the residency program.
- **Review descriptions of residency program and positions**: Evaluate whether the descriptions are consistent across all postings and align with the mission and goals of the institution and program.
- **Brainstorm desired characteristics**: Identify and define the attributes and qualities valued in a resident, ensuring these characteristics contribute to successful performance and align with and contribute to the institutional and program mission.
- **Determine assessment methods**: Develop strategies for evaluating the identified characteristics during the stages of the review process (i.e., Application Review, Screening Interview, and Full Interview).

#### 5.1.2 Applicant criteria identification and prioritization

The workbook includes separate tabs for each component of the EACM framework: Experiences, Attributes, Competencies, and Metrics. These tabs are intended to aid programs in further refining and adding to the characteristics brainstormed during the Preparation step. Users will:

1. Populate the selection criteria in each EACM tab by
  - Using the dropdown menu to select characteristics brainstormed in the Preparation tab that correspond to the EACM category. The text for the characteristics selected can also be freely edited on these tabs as needed.
  - Adding new criteria with free text entry.
2. Assign an importance level (Very Important, Important, Somewhat Important, Not Important) to each criterion. This ranking of importance will automatically sort and filter the criteria in the following steps. Criteria marked as “Not Important” will be filtered out in the following steps.
3. Determine whether each criterion is a hard requirement, that is criteria where an applicant will not proceed to the next stage of recruitment even if one criterion is not met.
4. Identify the stage(s) where characteristics will be evaluated: Application Review, Screening Interview, and/or Full Interview.

**FIGURE 3:**
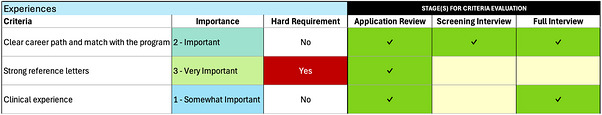
An example demonstrating use of an applicant criteria identification and prioritization EACM tab.

An example demonstrating use of the Applicant Criteria Identification & Prioritization EACM tabs is shown in Figure 3. This systematic process ensures that selection criteria are clearly identified and prioritized, which provides transparency and specificity for all faculty and staff participating in candidate evaluation.

#### 5.1.3 Final selection criteria

**FIGURE 4:**
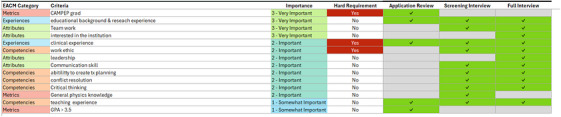
An example of the final selection criteria overview.

This section consolidates all selection criteria identified in the previous EACM tabs into a comprehensive list ranked by importance (Figure 4). This comprehensive overview will guide development for the rest of the holistic review process and can also help ensure that all team members involved are aligned on recruitment goals. Hard requirement designations and stages for criteria evaluation are also shown for reference. The list should encompass all criteria that will be evaluated and include criteria across all EACM categories. It is critical that the information in this section is reviewed for accuracy, balance, and completeness prior to proceeding with the workbook as the following sections will be populated based on this list. If changes are needed, return to the EACM tabs and edit the criteria there before continuing.

#### 5.1.4 Application review

The application review stage is a crucial component of the selection process because it serves as a pivotal point for candidacy. When applicants are excluded at this stage, the opportunity to reconsider their applications is permanently lost. Consequently, it is essential to conduct a thorough and fair review to ensure that all qualified candidates are given the opportunity to advance in the selection process.

The criteria selected for evaluation during the Application Review stage will automatically populate on this tab. To evaluate applications, the implementation committee will generate an anchored rubric for each of these criteria. An anchored rubric (Table 2) is a scoring tool that defines specific criteria with detailed benchmarks for a range of performance levels. Anchored rubrics are referred to as “anchored” because assessment scores are tied to these specific, predefined standards. These rubrics allow each institution to tailor the evaluation to align with their unique criteria and selection processes while providing a structured and consistent framework for reviewing and scoring applications, minimizing bias, and ensuring alignment with program goals.

**TABLE 2:** Example of an anchored rubric evaluating teamwork on a 5‐point scale that is “anchored” with detailed descriptions of specific standards that are tied to assessment scores.

Exceptional (5)	Moderate (3)	Low (1)
Actively fosters discussion and collaboration; ensures all team members are heard and able to contribute.	Contributes to discussions; supports contributions from other team members.	Limited contributions to discussions. AND/OR Limited support of other team members’ contributions.
Works effectively with a wide range of individuals from different backgrounds, including individuals with significantly different backgrounds than their own.	Works effectively with individuals from different backgrounds.	Limited experience working with individuals from different backgrounds. OR Unable to work effectively with individuals from different backgrounds.
Recognizes the importance of actively creating a culture and environment conducive to teamwork.	Recognizes the importance of having a culture and environment conducive to teamwork.	May or may not acknowledge the benefits of a conducive teamwork environment.
Takes initiative to establish, develop, and maintain a collaborative and collegial work culture and environment.	Contributes to maintaining a collaborative and collegial work culture and environment.	Does not actively work toward a collaborative and collegial work culture and environment.
Manages differences with/among teammates effectively; able to resolve differences and work together to come up with effective solutions.	Able to work around differences with/among teammates.	Limited experience with resolving differences among teammates. OR Unable to manage or resolve differences with/among teammates.
Provides specific examples where their teamwork met goals and directly resulted in measurable outcomes.	Provides examples where their team was able to meet goals.	Provides vague or limited examples of teamwork contributions. AND/OR
Clearly and thoughtfully articulates their role and contributions within the team.	Able to articulate their role within the team.	Unable to articulate their role within the team.

*Note*: In this example, anchors are provided for scores of 1, 3, and 5 points, which allows interviewers to infer scores in between these values, but programs can also choose to anchor their rubrics with descriptions for as many point values as deemed appropriate. The table in the workbook is set up for entry of up to 5 assessment levels, but programs can create scales with as many or as few points as appropriate for the criteria being evaluated. For the screening interview and full interview stages, the anchored rubrics should be developed specifically to assess answers to the questions selected.

For each criterion listed, the implementation committee would describe the standards that constitute performance at different levels. The table in the workbook is set up to allow up to 5 “anchors” for each criterion, but users can describe as many or as few as appropriate for the criteria to be evaluated.

#### 5.1.5 Screening interview and full interview

The two interview tabs, Screening Interview and Full Interview, operate analogously and will both be described in this section. Residency programs that do not conduct screening interviews can bypass the Screening Interview tab and proceed directly to Full Interview.

The criteria selected for evaluation during the Screening Interview and Full Interview stages will automatically populate on their respective sheets as a “Screening Interview Criteria Checklist.” The checklists track questions for each of the selection criterion to be evaluated. Under the “Question(s) Added” column, icons will indicate whether questions have been added for the criteria on your list. A red X indicates that the criterion has not been added to the interview question list; a yellow triangle indicates that the criterion has been added to the interview question list, but a question for the criterion has not been selected yet; and a green check indicates the criterion has been added to the interview question list, and that every instance has a question selected.

The “Selecting Screening Interview Questions” portion of the tab is used to select up to 10 interview questions for the Screening Interview and up to 30 questions for the Full Interview. A dropdown menu allow selection of criteria indicated for evaluation in each of the interview stages, which then allows selection of interview questions evaluating these specific criteria from the Question Bank (see 5.1.6 Question Bank). The same criterion can be selected more than once if the implementation committee opts for multiple questions evaluating a given criterion.

The implementation committee will then generate anchored rubrics specifically tailored toward evaluating the criteria in the context of the questions selected. Table 2 presents an example of an anchored rubric for assessing teamwork that could be used in an interview. This structure ensures that interviews probe relevant EACM selection criteria and maintains consistency in scoring across candidates and between interviewers.

#### 5.1.6 Question bank

Programs will use this tab to create a bank of standardized interview questions for assessing the EACM selection criteria that were identified as very important, important, or somewhat important. Implementation committees should enter at least one question for every criterion designated for evaluation in the interview stages and can add as many questions per criterion as desired for future use.

The workbook also includes an example question bank featuring behavioral questions from the AAPM Spring Clinical Meeting 2014, which users can refer to or pull questions from in developing their custom question banks.^15^ This resource provides flexibility while offering a starting point for crafting effective interview questions.

## 6 ENSURING EFFECTIVE IMPLEMENTATION

### 6.1 How to execute the implementation plan

When initially implementing holistic review and adapting the workbook described to your program, the first step is to assess the readiness of the program and reflect on the current culture. The concepts and goals of holistic review should be introduced and discussed. Once the program has a clear understanding of holistic review, an implementation committee should be formed.

The committee should represent the viewpoints of the key members within the program.^6^ Consider not only including all physicists involved in the program, but further broadening the team to include current residents, interprofessional staff members involved in training residents (e.g., physicians, dosimetrists, radiation safety officers, therapists, technologists), and experts in the hiring practice such as human resource specialists. Including the varied perspectives of relevant team members can result in generating criteria that represent a synthesis of the different aspects and priorities of your program and institution. Involving relevant department members also helps generate buy‐in and further interest in using the workbook during the phases of recruitment.

As the implementation committee uses the holistic review framework to identify and prioritize criteria, create assessment rubrics, and develop standardized interview questions, training on the holistic review framework and best practices in recruitment will be critical. Conduct initial training sessions on the goals of holistic review and how they apply to your program, as well as the framework components and examples applied to medical physics education programs. If not already performed, additional training on potential decision‐making biases, U.S. Equal Opportunity Commission (EEOC) rules on recruitment practices (if the program is in the United States), Canadian Human Rights Commission (CHRC, if the program is in Canada), and MedPhys Match rules (if applicable) will also be important.^16^, ^17^, ^18^ Being aware of the EEOC and MedPhys Match rules as applicable will be critical when generating interview questions to prevent the potential for discrimination and to avoid illegal questions. Studies have revealed high rates of potentially discriminatory questions being asked and MedPhys Match rules being infringed upon during medical physics residency interviews.^19^ While the MedPhys Match serves as a centralized system to pair applicants with residency positions, programs utilizing alternative independent or local recruitment pathways face identical structural challenges. Regardless of match participation status, the core tenets of the holistic review framework apply equally to all recruitment models. Regular training on these topics for all program staff involved in the interview process is recommended to help reduce the potential rule violations. SDAMPP also provides training presentations for medical physics educators which are available online.^20^

Implementing a holistic review process for program recruitment provides clear expectations for the admissions committee and interviewers. Tools to operationalize the holistic review approach are critical for implementation. One study^10^ used an A3 framework, a tool commonly used in quality improvement in which a problem is analyzed and solutions are implemented and monitored, using a templated single sheet of paper (hence the name A3 for an 11 × 17″ size paper).^21^ The Accreditation Council for Graduate Medical Education (ACGME) provides resources that programs can use to start the process.^8^ The workbook provided here has been adapted by a national group of medical physics educators and residency directors to provide a more relevant, practical tool for our field.

Holistic review during each step of the recruitment process, from determining applicant criteria to generating final post‐interview ranking lists, takes a group approach to decision‐making. However, reaching group consensus with all individuals involved can be difficult to achieve. As noted in other publications, there is no one universally correct method or system for evaluating candidates during the residency selection process.^22^ Therefore, objective, consistent, and fair decision‐making during this process can be challenging, depending on the individuals involved. One can use strategies for making the best possible decisions at each step of the process. Foster group decision‐making by establishing open, collaborative discussions in a safe environment.^6^ Empower all involved individuals to share their opinions, ensure that one single voice does not unduly dominate over the group, and limit influences from external individuals not involved in the recruitment process.^6^

### 6.2 An iterative approach to the implementation plan

Implementing an entirely new candidate review process can be a major undertaking requiring considerable time and effort. Some residency programs may be well‐positioned to perform a complete retooling at once, while other programs may need a few recruitment cycles to develop and deploy a holistic review model and overcome any impediments. One barrier may be navigating institutional requirements (e.g., Graduate Medical Education policies, hiring and recruitment policies, mandatory portals and software applications) and receiving approval to modify recruitment practices. Another challenge may be program inertia and a mindset that candidate reviews and interviews have “always” been done the same way. On an individual level, some faculty and admissions committee members may have a particular interview “style,” a favorite set of questions, or know how best to perceive “fit.” The last major challenge can be the time and effort required to learn, implement, and refine a new system. These are all reasonable challenges that can be managed to achieve the benefits of holistic review described in the Introduction.

Setting aside the residency programs that will try to adopt a holistic review approach all in a single admissions cycle, this section will focus on and provide a suggested roadmap for those programs that want a phased, multi‐cycle implementation. This roadmap has three suggested phases but can be expanded or contracted to each program's unique needs.

The first step is to compare a program's existing recruitment and admissions review process with the holistic review paradigm. If there is mostly overlap between the two with only minor process differences, then transitioning to a holistic review can be quick. However, if the processes are substantially different, either in overall degree or a few key steps are discordant, then more time and effort will be required to implement and get buy‐in.

#### 6.2.1 Year 1: Establishing the foundation

In the first year of a three‐year phased rollout, the focus is to establish what characteristics are important for candidates to possess, identify what criteria will be used to assess these characteristics, and verify that these characteristics are informed by or map onto overarching missions and goals. It is beneficial to have missions, values, and goals documented together and ensure that the program's marketing materials (e.g., job description, residency website) are consistent with these elements.

From the missions, values, and goals, draft a list of what characteristics are important for residents to succeed in the program. Develop this list with the EACM categories in mind and be open to critically evaluating any metrics or characteristics that have been used historically. For example, if having one peer‐reviewed publication is an expectation, consider what makes a peer‐reviewed publication so important. Is it the mere fact of having a publication, or is a publication a surrogate that demonstrates determination, teamwork, critical thinking, or something else? If a publication is a surrogate, are there other means to assess the underlying desired trait? It's also helpful to be aware that there are some characteristics that are valued by the institution but may not be apparent in the CV, personal statement, or letters of recommendation. For example, the candidate may take feedback exceptionally well, but by circumstance, this characteristic does not come through in the paper application files. Consider assessing those characteristics at different stages of the selection process, such as during screening or full interviews. Take note of these characteristics during the application review step, then, in the second year of implementation, adjust the stage of the selection process to assess specific characteristics. The goal of an anchored rubric is not to apply a single, rigid standard to all applicants, but to provide a framework for consistent evaluation of a desired attribute, accounting for an applicant's individual context. If the institution has a recruitment or hiring portal, it can be leveraged to avoid duplicate work. Often, these tools already have some characteristics and assessments that are mapped to institutional values, but they probably were not designed for a specific residency program.

Throughout the course of applicant evaluation, interviewing, and selection, keep this foundational work in mind. Change does not need to happen in this first year. Rather, observe if the whole review process is consistent with the mission, goals, and characteristics that were identified. At the end of the review process, update any notes or documents for use next year.

#### 6.2.2 Year 2: Refining and applying weightings and stages

In the second year, changes are starting to be made, and aspects of holistic review will be implemented. Update the program's marketing materials to be consistent and accurately reflect the type of candidate sought.

Revisit the characteristics and criteria (EACM) that were drafted in the first year. Verify that they are still accurate. Next, for each characteristic, a weighting must be applied, and a stage(s) of the recruitment process must be assigned for when to assess each characteristic. Assigning weights or priorities to each characteristic is necessary because some aspects will be absolutely required (e.g., the institution requires a terminal degree for a resident) while others are desirable but flexible (e.g., having passed ABR Part 1). This is another step where the holistic review process is tailored to each program's specific needs and goals. Lastly, there should be at least one identified stage of the applicant review process where each characteristic is assessed. Some components, especially metrics, can be sourced from the submitted application. Other EACM characteristics can be evaluated by contacting references, viewing an applicant's presentation, and asking interview questions. If a catalogue of questions asked by the program's interviewers does not already exist, during this year's interview stage, request each interviewer to submit a list of questions or question types that are most commonly asked. Having this information will be critical for the next phase.

Throughout the course of the applicant review this year, be mindful of EACM characteristics that were forgotten. This is also a good time to reflect on what stood out to reviewers about candidates that they believed to be strong to further refine and define the qualities within the rubric. At the end of the review process, update notes or documents for use next year.

#### 6.2.3 Year 3: Ensuring consistency and continuous improvement

The third year focuses on ensuring consistency in the interview process. One of the most challenging changes can be standardizing interview questions, as some interviewers may be resistant to giving up their preferred questions. Standardized questions offer numerous advantages for program directors, faculty interviewers, and candidates. For Program Directors, they will have a documented process that can easily be reported to institutional and accrediting bodies, and standardized questions can reduce the program's exposure and risk that an interviewer will ask an inappropriate question. For interviewers, having well‐established criteria and expectations makes candidate evaluation much simpler. The guesswork of what to ask and how to evaluate each interviewee can be minimized. For the candidates, it can be much more interesting, engaging, and informative about a program if each interviewer asks a different set of questions. Rather than rotating through and having the same repetitive, rehearsed conversation about themselves, how they found out about medical physics, what they hope to accomplish in residency, and what they intend to do afterwards, the candidates can address thought‐provoking questions that also convey what the program is looking for.

When implementing standardized questions, it requires talented interviewers to balance consistency with the ability to have a natural conversation and avoid making the interview process feel robotic. Some programs may choose to restrict interviewers to specific scripted questions, while other programs may allow for follow‐up questions and discussion.

When the selection stage is eventually reached, it may be challenging to trust the numerical ratings and results of the holistic review process, especially early on or if the results do not match the interviewer's impressions. This is a human process and is not strictly logical, so some inconsistency is acceptable. First, these ratings are modifiable and do not have to be rigidly adhered to, but exceptions and deviations from the ratings should be rare, well justified, and appropriately documented. Second, this may simply indicate the need to revise EACMs and reassess which stages and how they are evaluated. Save some time at the end of the selection meeting to discuss what went well and what did not. Did the candidate group that made it through the screenings and the interviews align with the program's recruitment goals? At the start of each year's recruitment cycle, reflect on whether any adjustments are needed based on the previous year's experiences.

### 6.3 When reality strikes: Challenges to implementation change

Even if programs are enthusiastic toward holistic review, careful planning and significant effort will be needed for implementation. Small changes to recruitment practices can be hard, and it is probable that implementing a holistic review involves large changes, so barriers and challenges may be encountered.

Holistic review is a relatively new concept. Some programs and faculty may not be familiar with the concept, and some may be hesitant to change recruitment strategies. Studies have found that barriers to implementing holistic review include a lack of consensus on the approach^23^ and a lack of faculty expertise.^24^ Strategies for building buy‐in and generating consensus and expertise include the following. The outcomes of holistic review in both undergraduate and graduate medical education are increasingly being reported, making it useful to reference data from other programs that have adopted this recruitment strategy. For example, one scoping review of holistic review implementation across Undergraduate Medical Education and Graduate Medical Education identified 33 articles and found that nearly all reported increases in the percentage of interviewed or selected applicants from underrepresented groups. Most programs paired holistic review with additional interventions—such as unconscious bias training or diversified search committees—which likely contributed to these outcomes.^25^ One's own program's past recruitment efforts may demonstrate a need for holistic review, so analyzing one's own internal data may prove helpful. The AAMC has multiple resources about holistic review and best practices for recruitment.^8^ In addition to the AAMC, there may be local experts, such as the institution's Graduate Medical Education office or Human Resources, who can provide assistance. Finally, joining forces with other residency programs within the same institution, such as the physician radiology or radiation oncology programs, or other medical physics residency programs, could assist in facilitating the implementation of holistic review. Effective communication will be critical when implementing the change to the holistic review.

The recruitment process entails multiple steps and a large amount of time and effort to thoroughly review applicants and make careful decisions. An assessment of the program's bandwidth and current clinical demands may reveal that the timing is not optimal for a sustained transformation. The move towards holistic review should be undertaken well in advance of the year in which the change will happen in order to give sufficient time for programmatic changes, especially in situations of limited bandwidth. The implementation team can also strategically choose which elements of the recruitment process to change so that the needed efforts are within current resource constraints. A program may want to start with a smaller scope, such as implementing a holistic review only during one step of the process or with a smaller group of individuals, then iteratively increase the scope of implementation during subsequent recruitment cycles.

The following table (Table 3) summarizes barriers and potential solutions to barriers and challenges when implementing a holistic review. As more programs implement holistic review, there will be more opportunities to share experiences and additional implementation strategies.

**TABLE 3:** Barriers and solutions for holistic review.

Barriers	Solutions
Lack of consensus on the approach	Communication Training
Lack of faculty expertise	Communication Training Join forces with local experts or other programs
Lack of time and resources	Use tools created by relevant organizations (SDAMPP, AAMC, etc.) Carefully select the scope and iterate over a longer time frame Establish an implementation committee
Hesitancy to change	Communication Use a team approach Data from local programs or from literature

## 7 RECOMMENDATIONS

The following recommendations summarize the specific actions that can be taken by residency programs to implement a holistic review approach aimed to enhance the effectiveness and fairness of the resident recruitment process:

1. **Generate interest and build buy‐in for implementing holistic review**: Highlight the value of a holistic approach, showcase data and success stories, engage key team members early, and proactively address concerns.
2. **Bring together varied perspectives**: Create teams composed of people with varied backgrounds, program/department roles, and viewpoints to reduce the influence of discrimination in the decision‐making process.
  - **Assemble an implementation committee**: Gather a team of individuals to create and oversee the creation of the holistic review framework. When assembling the implementation committee, consider including a wide range of stakeholders, such as faculty members, admissions staff, and current residents. This variety will ensure that multiple perspectives are represented, which can enhance the holistic review framework by incorporating various insights and experiences. Additionally, it fosters a sense of ownership and commitment to the process among all involved parties.
  - **Assemble a selection committee**: Gather a team of individuals to carry out the holistic review process created by the implementation committee. When assembling your selection committee, prioritize training and calibration sessions for all members to ensure a shared understanding of the holistic review criteria and process. This training should include discussions on implicit bias, evaluation standards, and the importance of a holistic approach from the institution, program director, human resources, and the graduate medical education office. By aligning the committee members on these key aspects, you can promote consistency in evaluations and foster a more equitable selection process.
3. **Provide Training**: Anyone involved in the residency recruitment process should receive training on unconscious bias mitigation and the implementation of the holistic review process to ensure that everyone has the tools and knowledge to implement these practices.
4. **Use the Medical Physics Holistic Review Workbook to help guide implementation**:
  - **Reflect on program mission and vision**: Determine if the program's description aligns with its stated mission and goals, brainstorm key characteristics and qualities valued in a resident, and develop strategies for evaluating the identified characteristics during the review process
  - **Define EACM selection criteria**: Establish clear guidelines on the experiences, attributes competencies, and metrics that align with the program's goals and mission. Next, assign importance level to each criterion, decide if it is a hard requirement, and determine at what stage of review the characteristic will be evaluated.
  - **Create anchored scoring rubrics**: Based on the EACM criteria identified, create standardized anchored scoring rubrics that outline specific criteria and performance indicators for evaluating candidates, ensuring consistent and objective assessment across all applicants and interviewees. Rubrics should be created for the application review, screening interview, and full interview steps.
  - **Create a list of standardized questions for a structured interview**: Develop a set of standardized questions to ask all candidates during the screening and full interviews to ensure consistent and objective evaluation. Programs can use questions from the pre‐populated question bank and/or add custom questions tailored for their needs.
5. **Foster group decision‐making**: Reaching a group consensus on final post‐interview ranking lists can be challenging, so it's important to establish open and collaborative discussions where individuals are empowered to share their opinions in a safe environment.
6. **Monitor and evaluate the process**: Continuously assess the effectiveness of the holistic review process by analyzing recruitment outcomes and incorporating feedback to address gaps and improve future recruitment cycles.

If implementing the entire process at once seems too overwhelming, programs can start with small changes, assess their impact, and refine the process based on feedback to build confidence before full implementation.

## 8 CONCLUSION

A holistic review process for residency program recruitment guided by the EACM model can help ensure the selection of candidates who possess not only strong academic foundations but also the experiences, attributes, and competencies needed for success in residency. However, implementing this can be challenging due to the lack of available resources, lack of existing standardization, and the large amount of time and effort that may be needed to make such a change. This report provides an overview of the core principles of holistic review and offers practical guidance for identifying and overcoming potential barriers to its successful implementation. Additionally, this report presents step‐by‐step actionable recommendations and includes a comprehensive workbook tool designed to help residency programs transition to a holistic review framework. By identifying and evaluating a broader range of qualifications that lead to success in clinical medical physics, holistic review will allow us to build a more dynamic and versatile workforce, ultimately strengthening both residency programs and the future of medical physics.

## AUTHOR CONTRIBUTIONS

All authors contributed to every part of this work: developing the idea and the approach, conducting the literature review, drafting and revising the manuscript, and ensuring accuracy and clarity. The corresponding author led these efforts. All authors have approved the final manuscript and consent to its submission to JACMP.

## CONFLICT OF INTEREST STATEMENT

The authors declare that there are no financial or non‐financial conflicts of interest related to this work.

## Supporting information




**Supporting File**: acm270649‐sup‐0001‐Data.zip.
